# Acute Pure Motor Cranial Polyneuropathy: A Rare Form of Guillain-Barré Syndrome

**DOI:** 10.7759/cureus.80079

**Published:** 2025-03-05

**Authors:** Kamal Haddouali, Mouad Ould Chhaiba, Hicham El Otmani, Mohammed Abdoh Rafai, Bouchra El Moutawakil

**Affiliations:** 1 Department of Neurology and Neurophysiological Explorations, Ibn Rochd University Hospital - Hassan II University, Faculty of Medicine and Pharmacy, Casablanca, MAR; 2 Research Laboratory on Diseases of the Nervous System, Neurosensory, and Handicap, Ibn Rochd University Hospital - Hassan II University, Faculty of Medicine and Pharmacy, Casablanca, MAR; 3 Genetics Laboratory, Ibn Rochd University Hospital - Hassan II University, Faculty of Medicine and Pharmacy, Casablanca, MAR

**Keywords:** cranial nerves palsy, cranial polyneuropathy, guillain-barré syndrome, oculo-pharyngeal variant, pure motor

## Abstract

Guillain-Barré syndrome (GBS) typically presents with ascending flaccid paralysis, but variants with exclusive cranial nerve involvement are rare. We report an atypical case of GBS with isolated motor cranial nerve involvement. A 19-year-old female with no significant medical history presented with bilateral ptosis and diplopia, which progressed over 10 days to bilateral facial paresis, lingual and masseter weakness, and severe dysphagia. Neurological examination revealed symmetric involvement of cranial nerves III, IV, VI, VII, IX, and XII, without sensory or tendon reflex abnormalities. Nerve conduction studies showed absent motor responses in the facial nerve and blink reflex, along with repetitive distal compound muscle action potentials, initially suggestive of congenital myasthenia. These findings normalized after the discontinuation of acetylcholinesterase inhibitors. Assessment of the neuromuscular junction was normal. Electromyography revealed neurogenic changes in the masseters and genioglossus. Brain MRI and cerebrospinal fluid analysis were normal, and infectious serologies and autoimmune screening were negative. The patient was diagnosed with the oculo-pharyngeal variant of GBS. Despite treatment with intravenous immunoglobulin and five sessions of plasmapheresis, no significant improvement was observed in the short term. At six months, the patient continued to experience persistent ophthalmoparesis, dysphagia, and facial amyotrophy, requiring gastrostomy placement. This case highlights the rare oculo-pharyngeal variant of GBS, characterized by exclusive cranial nerve involvement, which poses diagnostic challenges and often leads to poor functional outcomes. Early recognition and prompt treatment are crucial to improving prognosis.

## Introduction

Guillain-Barré syndrome (GBS) is an acute inflammatory disorder that typically presents with ascending paralysis within four weeks, characterized by flaccid weakness and areflexia [1]. Cranial nerve involvement is observed in 15% to 75% of cases, often as part of a typical clinical presentation. Other forms of GBS, such as Miller Fisher syndrome (MFS) and pharyngeal-cervical-brachial (PCB) syndrome, involve cranial nerves more prominently. However, isolated cranial nerve palsy is a rare manifestation of GBS and is occasionally reported as an oculo-pharyngeal variant, with few cases described in the literature [2]. This form is often referred to as idiopathic cranial polyneuropathy. When the involvement is purely motor, it presents significant diagnostic challenges, particularly when distinguishing it from myasthenia gravis and other pathologies such as botulism or skull base tumors. Although the exact pathophysiology remains unclear, axonal damage and treatment delays contribute to poor functional outcomes. We present a case of this rare GBS variant, focusing on the cranial nerves diffuse pure motor involvement, highlighting its diagnostic complexity and therapeutic challenges.

## Case presentation

A 19-year-old female with no notable medical history presented with bilateral ptosis and binocular diplopia, which progressed over 10 days without fluctuation or pain. Over time, bilateral facial paresis and lingual and masseter weakness developed, resulting in severe dysphagia. By the 21st day, no new symptoms had emerged. At admission, physical examination revealed bilateral ophthalmoplegia without involvement of the photomotor reflex, severe facial diplegia, lingual immobility and atrophy, abolition of the gag reflex, and paralysis of the masticatory muscles with masseter atrophy. These clinical signs indicated the motor involvement of cranial nerves III, IV, VI, VII, IX, and XII. Muscle strength in the limbs, tendon reflexes, and sensory testing were all normal. A brain MRI showed no abnormalities, particularly no hypersignals or post-injection enhancement involving the cranial nerves. Cerebrospinal fluid analysis revealed normal findings (3 cells/mm³ on cytology, protein at 0.4 g/L, and glucose at 2.5 mmol/L).

**Figure 1:**
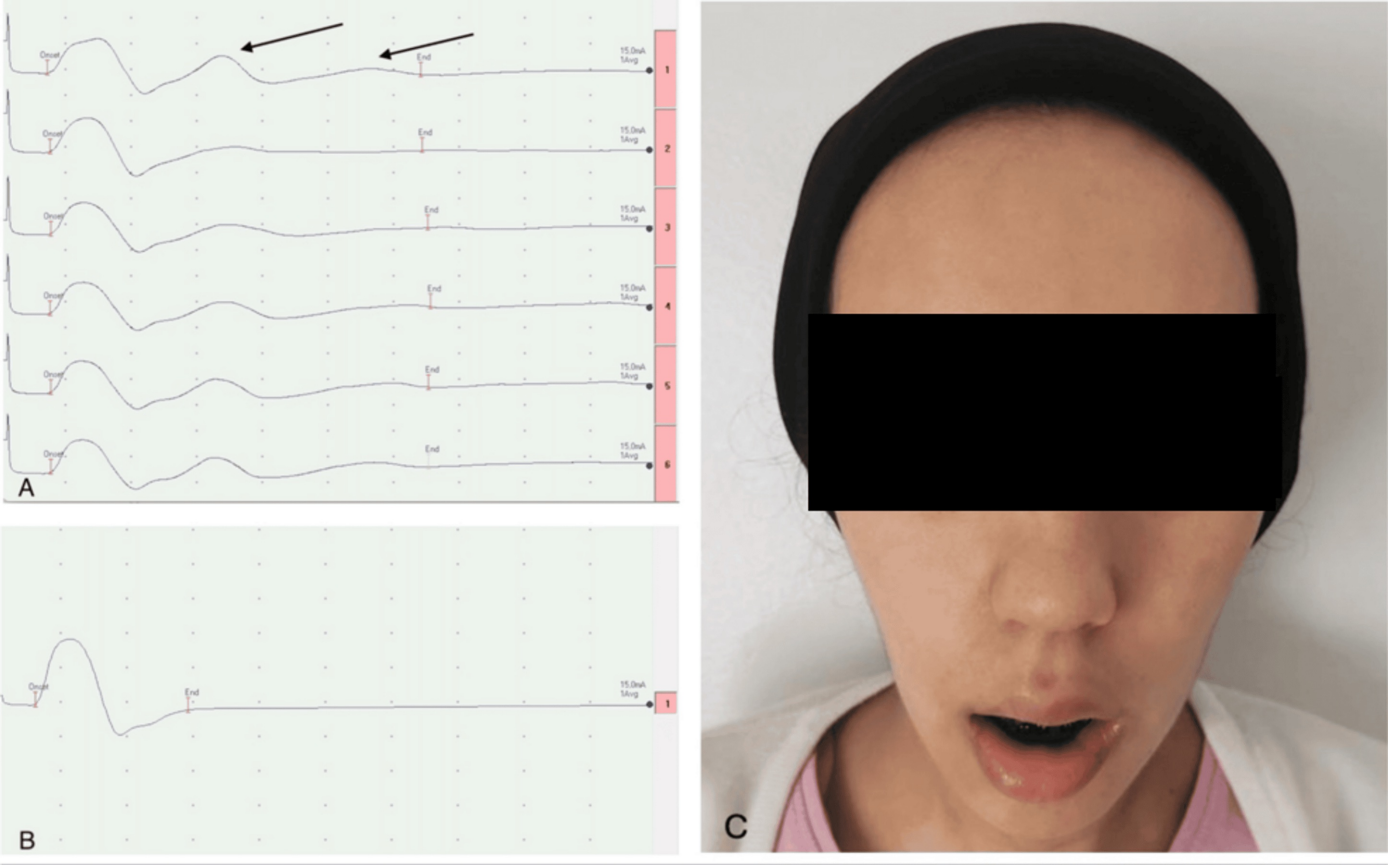
Clinical and electrophysiological features of the patient A: NCS (5mV-5ms/div): Repetitive dCMAP of the right median nerve (arrows) due to acetylcholinesterase inhibitors therapy, disappearing after the short exercise test and reappearing around the 20th second (fourth stimulation). B: Normal morphology of dCMAP of the right median nerve performed 48 hours after discontinuation of acetylcholinesterase inhibitors therapy. C: Clinical status of the patient at six months, showing inability to perform tongue protrusion, bilateral facial paresis, masseter amyotrophy, and permanent jaw dropping. NCS: nerve conduction studies, dCMAP: distal compound muscle action potentials

Nerve conduction studies (NCS) revealed absent motor responses in the facial nerve and blink reflex and repetitive distal compound muscle action potentials (dCMAP) in the median and ulnar nerves following single stimulation. The repetitive dCMAP disappeared after a brief exercise test and reappeared after approximately 20 seconds (Figure 1A), initially suggesting an acute decompensation of congenital myasthenia. However, it was later determined that the patient was taking acetylcholinesterase inhibitors, which caused this abnormal dCMAP response. After discontinuing the acetylcholinesterase inhibitors, normal dCMAP morphology was restored within two days (Figure 1B). Assessment of the neuromuscular junction through low-frequency (3 Hz) repetitive nerve stimulation of all four limbs and the face was normal. Electromyography revealed fibrillation potentials in the masseters, genioglossus, and chin muscles, consistent with neurogenic changes. Testing for anti-acetylcholine receptor antibodies, anti-muscle-specific kinase antibodies, anti-ganglioside antibodies, infectious serologies, and systemic autoimmune inflammatory markers was negative.

Based on these clinical and electrophysiological findings, the patient was diagnosed with an oculo-pharyngeal variant of GBS, characterized by exclusive motor cranial nerve involvement. The patient was initially treated with intravenous immunoglobulin (IVIg) at a dose of 1 g/kg over two days on the 10th day of illness, suspecting an acute decompensation of myasthenia gravis. However, after confirming the final diagnosis on the 21st day, she underwent five sessions of plasma exchange, with no significant improvement observed in the short term. At the six-month follow-up, despite undergoing orthoptic and orthophonic therapy, the patient continued to experience ophthalmoparesis, lingual paresis, dysphagia, and diffuse facial amyotrophy, ultimately requiring gastrostomy placement (Figure 1C).

## Discussion

Although GBS typically presents with flaccid ascending tetraparesis and areflexia, cranial nerve involvement is common, occurring in approximately two-thirds of cases. "Cranial" or "mesocephalic" GBS, first described by Guillain in 1937, affects the cranial nerves while sparing the limbs and represents less than 5% of all GBS cases [3]. The 2023 guidelines from the European Academy of Neurology/Peripheral Nerve Society include clinical variants involving cranial nerves, such as MFS, PCB syndrome, and bifacial weakness with limb paresthesia. However, they do not recognize a subtype with exclusive cranial nerve involvement [4]. Wakerley and Yuki proposed classifying isolated acute cranial polyneuritis as a distinct GBS subtype [5].

In the literature, approximately 10 cases of pure motor cranial polyneuropathy have been reported. These cases, including the one presented here, often result in poor functional outcomes, likely due to the axonal pattern observed in NCS and delays in diagnosis and treatment [6]. Unlike MFS and PCB, where albuminocytologic dissociation is a hallmark feature, our patient did not exhibit this finding. This contrasts with the study by Wang et al., who observed albuminocytologic dissociation in half of the cases within the first week and in all cases within two to three weeks [7].

According to several guidelines, the standard IVIG dose for GBS is 2 g/kg, administered over two to five days. Therefore, the 1 g/kg dose administered to our patient, based on an initial misdiagnosis, was insufficient, which may have contributed to suboptimal results [8]. Despite undergoing five sessions of plasma exchange, the patient has shown no significant improvement, which is consistent with the variable responses commonly seen in cranial nerve forms of GBS [9].

The exact pathophysiological mechanisms of this rare variant remain unclear. However, similarities with MFS and PCB have been suggested, particularly the involvement of anti-ganglioside antibodies, such as anti-GM1 or anti-GD1a antibodies, which are more commonly associated with axonal forms of GBS [10]. Further research is necessary to better understand the pathophysiology of this rare GBS variant and to optimize treatment strategies.

## Conclusions

This case highlights a rare GBS variant characterized by exclusive motor cranial nerve involvement. Early recognition through comprehensive clinical and electrophysiological analysis is crucial to prevent delays in diagnosis and treatment, which can significantly impact prognosis. Given this GBS variant's rarity and potential severity, further research is needed to understand its pathophysiology better and optimize management strategies.
